# Medication-overuse headache: a widely recognized entity amidst ongoing debate

**DOI:** 10.1186/s10194-018-0875-x

**Published:** 2018-07-13

**Authors:** Nicolas Vandenbussche, Domenico Laterza, Marco Lisicki, Joseph Lloyd, Chiara Lupi, Hannes Tischler, Kati Toom, Fenne Vandervorst, Simone Quintana, Koen Paemeleire, Zaza Katsarava

**Affiliations:** 1 Headache Group, Department of Basic and Clinical Neuroscience, King’s College London, and NIHR-Wellcome Trust King’s Clinical Research Facility, King’s College Hospital, Denmark Hill, London, SE5 9PJ UK; 2 Department of Neuroscience, St. Agostino Estense Hospital, University of Modena and Reggio Emilia, via P. Giardini 1355, 41100 Modena, Italy; 3 Headache Research Unit, Université de Liège, Liège, Belgium; 4 Headache Research-Wolfson CARD, King’s College London, London, UK; 5 Headache Centre, Careggi University Hospital, Health Sciences Department, University of Florence, Viale Pieraccini 6, 50139 Florence, Italy; 6 Department of Neurology, Medical University Innsbruck, Innsbruck, Austria; 7 Department of Neurology, Tartu University Clinics, Tartu, Estonia; 8 Estonian Headache Society, Tartu, Estonia; 9 Department Neurology, UZ Brussel, Jette, Belgium; 10 Headache Center, Department of Medicine and Surgery, University of Parma, Parma, Italy; 11 Department of Neurology, Ghent University Hospital, Corneel Heymanslaan 10, B-9000 Ghent, Belgium; 12 Evangelical Hospital Unna and University of Duisburg-Essen, Duisburg, Germany

**Keywords:** Medication-overuse headache, Review, Nosology

## Abstract

Medication overuse in primary headache disorders is a worldwide phenomenon and has a role in the chronification of headache disorders. The burden of disease on individuals and societies is significant due to high costs and comorbidities. In the Third Edition of the International Classification of Headache Disorders, medication-overuse headache is recognized as a separate secondary entity next to mostly primary headache disorders, although many clinicians see the disease as a sole complication of primary headache disorders. In this review, we explore the historical background of medication-overuse headache, its epidemiology, phenomenology, pathophysiology and treatment options. The review explores relevant unanswered questions and summarizes the current debates in medication-overuse headache.

## Background

Overuse of symptomatic medication is a common problem in patients with primary headache syndromes [1, 2]. Headache syndromes such as migraine or tension-type headache cause painful experiences and significant disability in patients. The use of analgesics is therefore justifiable when correctly utilized. For more than 50 years, clinicians have recognized and reported on headache chronification occurring during a period of frequent use of analgesics. The underlying consensus for the entity of medication-overuse headache (MOH) consists of a deterioration of a pre-existing headache syndrome whilst overusing one or several types of acute painkilling treatments. MOH is widely accepted and recognised in the neurological and headache community nowadays, although the entity keeps raising important questions. Debates on the pathophysiological mechanisms, definitions of overuse and the nosology of MOH are ongoing. This review presents the current state of literature and knowledge on MOH. It provides an overview of the history, clinical features, epidemiology of MOH, an update on the current understanding of the underlying neurobiological mechanisms and treatment, before discussing the key topics in the controversies surrounding MOH.

## MOH in historical perspective

The first descriptions of MOH date back to 1930s, when multiple authors started to associate prolongation of migraine with ergotamine-overuse [3–8]. Chronic headache following overuse of ergotamine was clearly defined by Peters and Horton in 1951 [9]. They reported on 52 migraine patients who developed daily headache after daily use of ergotamine and noted improvement after the drug was stopped. The same authors published their withdrawal protocol in 1963 [10]. The first ergotamine withdrawal protocols were proposed independently by Graham, Friedman and Lippmann in 1955 [3, 6–8]. In the 1970s, multiple authors wrote on the association between overuse of mixed analgesics, including those based on ergotamine, barbiturates and codeine, and headache progression [11, 12]. In 1982, Mathew et al. outlined that overuse of analgesics contributed to the transformation of episodic migraine (EM) into daily headaches and a few years later the same group introduced the term “transformed or evolutive migraine” to describe the entity [3, 13, 14].

The first edition of the International Classification of Headache Disorders (ICHD) was published in 1988 which introduced the term “drug-induced headache”. It also introduced and specified the entities “ergotamine-induced headache”, “analgesics abuse headache” and “other substances” [15]. This was based on the experience with overuse of analgesics and ergots only. After the introduction of triptans, it became clear that this class of drugs could also induce headache deterioration if used excessively [16–18]. In 1994 Silberstein et al. proposed criteria for “transformed migraine”, since transformation of EM to daily or almost-daily head pain (> 15 days/month) was associated with medication overuse [3, 19].

The term “medication-overuse headache” was first introduced in the second edition of the ICHD in 2004 [20]. It also defined MOH subtypes induced by simple analgesics, combination-analgesics, ergots, triptans and opioids. The diagnostic criteria included a mandatory prerequisite that the headache syndrome resolved or reverted to the previous pattern within 2 months after discontinuation of the overused drug. This caused the entity of definite MOH to be diagnosed retrospectively and more difficult to handle in clinical practice [20]. The criterion was changed in 2006 when a board of experts published revisions by consensus and introduced a  broader concept of MOH, in which the diagnosis was based on headache frequency (equal to or greater than 15 days/month) and overuse of headache medication, but did not require the headache to improve after withdrawal [21]. This criterion was omitted again in the latest and current Third Edition of the International Classification of Headache Disorders (ICHD-3) [22].

## Current definitions

In ICHD-3, chronic headache syndromes are defined by expert consensus as headache disorders that share characteristics with pre-existing headache syndromes, occur for a certain amount of time (at least 3 months in e.g. chronic tension-type headache (CTTH), chronic migraine (CM); or at least 1 year in e.g. chronic trigeminal autonomic cephalalgia (TAC)) and have an additional time-criterion (e.g. headache days per month in CTTH and CM, or the absence of remissions for more than 3 months in TAC’s). MOH is found in ICHD-3 under subsection 8.2 as a chronic headache disorder secondary to a pre-existing headache syndrome. It is stipulated as a consequence of regular overuse of drugs for the acute treatment of headache. To establish the diagnosis, patients have to use symptomatic headache medication on more than 10 or more than 15 days per month, depending on the drug class, for more than 3 months. MOH has 8 subforms – MOH induced by ergotamine, triptans, analgesics including paracetamol, aspirin and other non-steroidal anti-inflammatory drugs (NSAIDs), opioids, combination analgesics, unspecified multiple drug classes and others (Table 1) [22].

**Table 1:** International Classification of Headache Disorders Third Edition (ICHD-3) Criteria for Medication-Overuse Headache (MOH) [22]

8.2 Medication-overuse headache (MOH) A. Headache occurring on ≥15 days/month in a patient with a pre-existing headache disorder B. Regular overuse for > 3 months of one or more drugs that can be taken for acute and/or symptomatic treatment of headache C. Not better accounted for by another ICHD-3 diagnosis. 8.2.1 Ergotamine-overuse headache A. Headache fulfilling criteria for 8.2 Medication- overuse headache B. Regular intake of ergotamine on ≥10 days/month for > 3 months. 8.2.2 Triptan-overuse headache A. Headache fulfilling criteria for 8.2 Medication- overuse headache B. Regular intake of one or more triptans,1 in any formulation, on ≥10 days/month for > 3 months. 8.2.3 Non-opioid analgesic-overuse headache 8.2.3.1 Paracetamol (acetaminophen)-overuse headache A. Headache fulfilling criteria for 8.2 Medication- overuse headache B. Regular intake of paracetamol on ≥15 days/ month for > 3 months. 8.2.3.2 Non-steroidal anti-inflammatory drug (NSAID)- overuse headache A. Headache fulfilling criteria for 8.2 Medication- overuse headache B. Regular intake of one or more non-steroidal anti-inflammatory drugs (NSAIDs) (other than acetylsalicylic acid) on ≥15 days/month for > 3 months. 8.2.3.2.1 Acetylsalicylic acid-overuse headache A. Headache fulfilling criteria for 8.2 Medication- overuse headache B. Regular intake of acetylsalicylic acid on ≥15 days/ month for > 3 months. 8.2.3.3 Other non-opioid analgesic-overuse headache A. Headache fulfilling criteria for 8.2 Medication- overuse headache B. Regular intake of a non-opioid analgesic other than paracetamol or non-steroidal anti-inflammatory drugs (including acetylsalicylic acid) on ≥15 days/month for > 3 months. 8.2.4 Opioid-overuse headache A. Headache fulfilling criteria for 8.2 Medication- overuse headache B. Regular intake of one or more opioids on ≥10 days/month for > 3 months. 8.2.5 Combination-analgesic-overuse headache A. Headache fulfilling criteria for 8.2 Medication- overuse headache B. Regular intake of one or more combination-analgesic medications on ≥10 days/month for > 3 months. 8.2.6 Medication-overuse headache attributed to multiple drug classes not individually overused A. Headache fulfilling criteria for 8.2 Medication- overuse headache B. Regular intake of any combination of ergotamine, triptans, non-opioid analgesics and/or opioids on a total of ≥10 days/month for > 3 months without overuse of any single drug or drug class alone. 8.2.7 Medication-overuse headache attributed to unspecified or unverified overuse of multiple drug classes A. Headache fulfilling criteria for 8.2 Medication- overuse headache B. Both of the following: 1. regular intake of any combination of ergotamine, triptans, non-opioid analgesics and/or opioids on ≥10 days/month for > 3 months 8.2.8 Medication-overuse headache attributed to other medication A. Headache fulfilling criteria for 8.2 Medication- overuse headache B. Regular overuse, on ≥10 days/month for > 3 months, of one or more medications other than those described above, 1 taken for acute or symptomatic treatment of headache.	

Although considered a general rule in the past, it is now well stated in the classification that MOH usually, but not invariably, resolves once the overuse is stopped [22, 23]. As with all secondary headache syndromes in ICHD-3, there is no longer a necessary requirement of remission or substantial improvement of the underlying causative disorder for the diagnosis to be made. Therefore, when MOH is confirmed by using the medical history of the patient, a two-fold diagnosis is made: the first one entailing the primary headache syndrome that resulted in drug overuse, the second one MOH [23].

## Epidemiology

The prevalence of chronic headache is 4% to 5%, with an incidence of 3% per year [24, 25]. The incidence of new-onset CM in patients with EM is around 2.5% per year [24, 26]. Even higher incidence rates up to 14% were reported from a tertiary centre [27]. Prevalence rates for MOH in the general population level are situated between 1 and 2%, with a range between 0.5% and 7.2% [28]. The highest prevalence has been shown in Russia (7.2%) [29]. Knowledge about prevalence and socio-economic burden in lesser developed countries has been very limited for a long time, although studies have been published lately for prevalence in Africa (Zambia 7.1%; Ethiopia 0.7%), Latin America (Brazil 1.4%, Colombia 4.3%) and Asia (Korea 0.5%; China 0.6%) [30–35] . MOH is estimated to affect around 63 million people worldwide [35–37]. The prevalence of medication overuse is higher in studies from headache specialist centers, with numbers ranging from 30% to 50% of patients [38–40].

A systematic review of epidemiological studies found that MOH is most common among middle-aged adults from 30 to 50 years of age, and predominant in females in the majority of studies. The male to female ratio is around 1 to 3–4 [28, 36, 41–43]. Among U.S. children and adolescents, the prevalence of CM was found to be 0.79% if medication overuse was excluded, and 1.75% if it was included [44, 45]. Prevalence of MOH was greater in girls than boys [44]. Furthermore, between 21% and 52% of pediatric patients with chronic headache met the criteria for MOH [45, 46]. Worldwide, the prevalence of MOH in pediatric samples was 3.3%, 0.3%, 0.5% and 1.6% in Italy, Taiwan, Norway and Canada respectively [37, 47–50]. In the elderly population, studies from multiple headache centers found that around 35% of patients older than 64 years were overusing medication [51, 52]. Reports on prevalence of MOH in specific populations and minorities have been published. In Europe, certain minorities or ethnic groups, such as first-generation migrants, show higher than expected rates of MOH. Potential explanations for these findings include socioeconomic (e.g. use of healthcare), biological (e.g. genetic) or cultural reasons (e.g. language barriers) [53].

The burden of disease for MOH has been shown to be a worldwide problem. The disorder causes important negative social and economic effects in both rich and poor countries. Mean per-person annual costs were calculated at €3561 for medication overuse [54]. Not only economic factors, but also psychological and physical disability of chronic headache and MOH needs to be considered. The global campaign “Lifting the Burden” has contributed to the acquisition of new data and to the promotion of accurate epidemiological methods all over the world [55, 56]. In the most recent issue of the Global Burden of Disease (GBD) in 2016, migraine became the second largest cause of disability, mainly because MOH was considered a sequela of migraine and tension-type headache [57].

## Risk factors

Medication overuse was found to be an important risk factor for chronification of primary headaches [58]. A systematic review analysed twenty-nine studies and found differences in the risk of developing MOH and the type of used drug. The risk was lowest for triptans (relative risk (RR) 0.65) and ergotamine (RR 0.41) compared to combined analgesics. Triptans and ergotamine containing drugs were found more favorable when compared to opioids [59]. This is in line with Bigal et al. who reported that people using medication containing barbiturates or opiates had a two-fold higher risk of developing chronic headache than patients using single analgesics or triptans. In this study, NSAIDs were protective against developing chronic headache at low to moderate level of monthly headache days, but were associated with an increased risk of developing chronic headache in patients with a high level of monthly headache days (more than 10 days per month) [26].

An important risk factor for the development of MOH is predisposition for migraine or tension-type headache as an underlying biological trait. Migraine is the most common pre-existing headache disorder complicated by MOH. Other pre-existing headache disorders can be complicated by MOH as well, such as tension-type headache or cluster headache [60]. Paemeleire et al*.* investigated the presence of MOH in patients suffering from cluster headache and found this complication only in patients also suffering with migraine or having at least a family history of migraine [61]. In addition, the clinical experience shows that the majority of patients suffering from cluster headache do not complicate into MOH although overuse of sumatriptan injections can lead to increased frequency of cluster attacks [62]. Patients with other chronic pain disorders who overuse painkillers for non-cephalic pain conditions do not seem to acquire chronic headache, unless they have a pre-existing history of a primary headache disorder [63, 64].

In a large prospective population-based study, Hagen et al studied 25.596 patients who did not suffer from chronic daily headache at baseline but had MOH 11 years later (*n* = 201, 0.8%) [65]. In this study, the following risk factors were found to be associated with the development of MOH: regular use of tranquilizers (odds ratio (OR) 5.2, 95% confidence interval (CI) 3.0–9.0), combination of chronic musculoskeletal complaints, gastrointestinal complaints and Hospital Anxiety and Depression Scale (HADS) score > = 11, physical inactivity (defined as > = 3 h hard physical activity/week), and smoking (daily vs. never). Furthermore, migraine was a stronger risk factor for MOH than nonmigrainous headache. A strong association was found for a high-frequency headache defined as 7–14 days/months compared to absence of headache days. Non-modifiable risk factors for MOH were age younger than 50, female gender and low level of education. Interestingly, the authors found several risk factors for MOH (e.g. smoking, inactivity) that were not found to increase the risk for chronic daily headache without the overuse of analgesics. Therefore, the authors concluded that both entities might be pathogenetically distinct [65]. Lastly, Cevoli et al. detected a more than threefold increased risk of MOH if a family history of MOH or other substance abuse, such as drug or alcohol abuse, was present [66].

## Clinical features of MOH

A comprehensive medical history, clinical examination and the use of internationally accepted criteria and guidelines are the required tools for the diagnosis of MOH. A confirmatory diagnostic test for MOH is currently not available. The headache phenotype of MOH may be indistinguishable from other forms of chronic daily headache. Moreover, the ICHD-3 criteria do not stipulate MOH-specific clinical features (such as headache characteristics or associated symptoms). Awareness for potential secondary headache syndromes is required and ‘red flags’ have to be searched for in order to the avoid a false-positive diagnosis of MOH in escalating headache disorders, some of which may require medical imaging or lumbar puncture. In practice, an in-depth enquiry of headache types, frequency and especially drug use is always mandatory, as overuse of ergotamine, triptans, NSAIDs, opioids, or analgesic combinations entail different prognostic properties [24, 67].

## Comorbidities

Comorbidity is the simultaneous existence of two or more different medical conditions. Comorbidities occur by chance, or by more than chance, suggesting a potential association, causality, common aetiological factors or common pathophysiological processes. In the field of MOH, these terms are often difficult to appoint although researchers have found multiple associations.

Psychiatric comorbidities in MOH are frequent and have been studied extensively since the earliest descriptions of patients with MOH [68]. MOH and mood disorders such as anxiety and depression are thought to be comorbid disorders by more than chance [13, 69–71]. In the BIMOH study, a prospective interventional study, Hospital Anxiety and Depression Scale (HADS) scores were collected in patients with MOH (before and after a brief intervention) and controls. MOH patients were found to show significantly higher HADS scores for anxiety [72]. In the “COMOESTAS” trial, using HADS, 40.0% of MOH patients fulfilled the criteria for depression and 57.7% for anxiety [73]. The “Eurolight” trial, a cross-sectional study in the adult population of ten European Union countries, found similar results. The association was even stronger compared to a group of patients with migraine without overuse [74].

In the Sodium Valproate in Medication Overuse Headache Treatment (SAMOHA) study, a more extensive screening for psychopathological comorbidities was performed in MOH patients in comparison to patients with EM and healthy controls [71]. The rate of moderate to severe anxiety in MOH was significantly higher compared to EM patients and healthy controls. Values on the Leeds Dependency Questionnaire were significantly higher in MOH patients compared to EM patients, which indicates a greater susceptibility to drug dependency. When looked at the number of psychiatric disorders, MOH patients were more likely to have multiple psychiatric comorbidities.

An association between clinically relevant obsessive-compulsive disorder (OCD) and MOH was demonstrated [71]. Around 30% of MOH patients are estimated to show clinical features of subclinical OCD on neuropsychological evaluation. Subclinical OCD may be an additional risk factor for headache chronification [75, 76]. Also, MOH can be associated to substance-related disorder spectrum, moreover since MOH and dependence share common neurobiological pathways, although MOH patients do not share common personality characteristics with drug addicts [77, 78].

For metabolic disorders, a couple of studies from North America on obesity found an increased risk of developing chronic headache, although in the European study by Hagen et al. no such association found [69, 79, 80]. In a Chinese cohort, an association between MOH and metabolic disturbances such as obesity and hypertension was shown in female patients [81]. Recent data on smoking, physical inactivity and obesity provided by a Danish cross-sectional analysis confirmed an association between MOH and those metabolic derangements although causality could not be proven [82]. In children, the association between obesity and chronic headache has been shown in observational studies, but the link with medication overuse is unclear [83, 84]. Lastly, patients with chronic headache and MOH present a high prevalence of sleep complaints [85].

## Pathophysiology

**Fig. 1:**
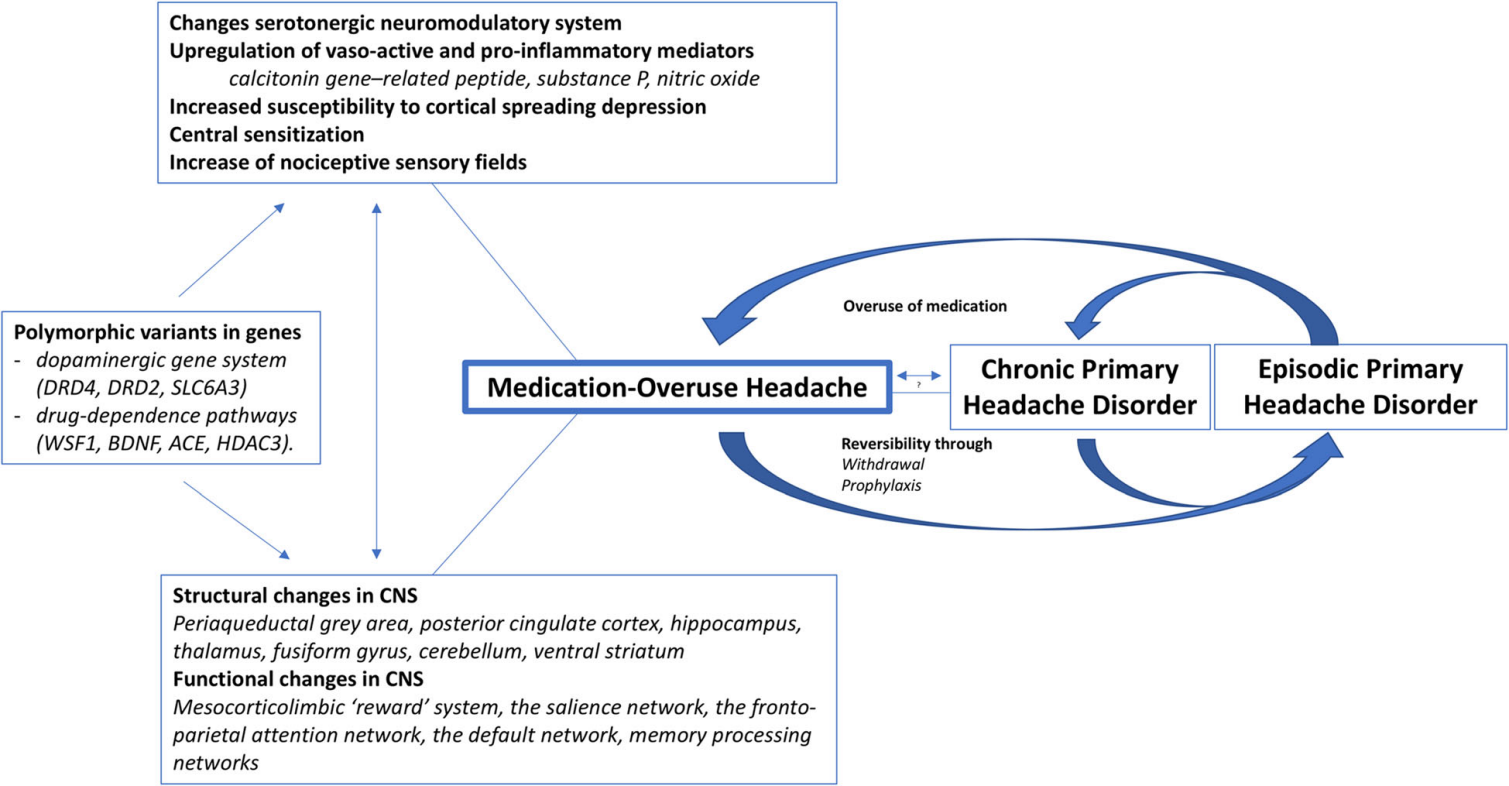
Current understanding of the pathophysiology of medication-overuse headache (MOH). The knowledge on the pathophysiology of MOH involves conversion from and reversion to primary headache disorders, showing changes in physiological processes, functional connectivity and structural changes of the central nervous system, in patients with underlying genetic susceptibility. Abbreviations: MOH: medication-overuse headache; CNS: central nervous system

A complete understanding of the pathophysiology of MOH currently does not exist [86–88]. Although the clinical aspects of MOH seem to be ambivalent, there is evidence for specific neurobiological aspects in MOH-models. Animal studies, genetic studies, structural and functional neuroimaging, and electrophysiological analyses have added to the current knowledge on the pathophysiology of MOH (Fig. 1).

Animal studies have shown changes in multiple physiological processes in the central nervous system (CNS) after repetitive administration of analgesics. Chronic sumatriptan exposure produces long-lasting increased susceptibility to evoked cortical spreading depression (CSD) due to lower threshold [89–91]. Upregulation of vaso-active and pro-inflammatory mediators such as calcitonin gene–related peptide (CGRP), substance P, and nitric oxide synthase were found in trigeminal ganglia [92, 93]. An expansion of the receptive nociceptive field, a decreased nociceptive threshold and decreased noxious inhibitory control have been reported [91, 94]. Furthermore, chronic exposure to analgesics was found to increase the excitability of neurons in the central nucleus of the amygdala, which may conceptualise the development of anxiety or depression in patients with MOH [95].

The serotonergic modulating system is presumably affected by chronic analgesic use, resulting in neuronal hyperexcitability, enhanced CSD and trigeminal nociception, caused by increased expression of pro-nociceptive serotonin 2A (5HT-2A) receptor binding sites and a decrease in the production of serotonin in the CNS [96–98]. In analogy to the findings in animals, an upregulation of 5HT-2 receptors on platelet membranes during analgesic abuse and lower platelet levels of serotonin were found, probably caused by suppressed serotonin transport [99].

Genetic studies have been performed in MOH although high-quality evidence for genetic traits is currently lacking. In a recent systematic review, Cargnin et al. described candidate polymorphic variants in genes of the dopaminergic gene system (DRD4, DRD2, SLC6A3), and genes related to drug-dependence pathways (WSF1, BDNF, ACE, HDAC3). The authors concluded that these traits are potential risk factors for MOH susceptibility or determinants of monthly drug consumption [100–107].

Research shows that central sensitization has a major role in the pathophysiology of MOH [88, 108]. Using somatosensory evoked potentials comparing cortical responses in MOH patients with responses in healthy volunteers and episodic migraineurs, hypersensitivity (a sign of central sensitization) and hyper-responsiveness of the cerebral cortex were shown in MOH patients as potential markers of altered functioning. The authors suggested that the somatosensory cortex in MOH patients is somehow “locked” in a kind of pre-ictal state [109, 110].

More recently, a cohort of MOH patients was followed during a 12 month period, evaluating central sensitization through pain-perception assessment. The authors found evidence of central sensitization at baseline, but most importantly, the study permitted to expose the slow progression towards normalization of sensory processing after detoxification during the extended follow-up window. This adds to the importance of detoxification and observation after withdrawal in order to prevent relapses [111].

Over the last decade, imaging studies have increased the knowledge of structural alterations and physiological events in MOH. Structural imaging studies performed by separate groups have found increased gray matter volume in following areas: periaqueductal gray (PAG) area, posterior cingulate cortex, hippocampus, thalamus, fusiform gyrus, cerebellum and ventral striatum [87, 88, 112]. Less volume was found in the orbitofrontal cortex (OFC), anterior cingulate cortex, left middle occipital gyrus, insula and precuneus [87, 88, 112]. These structures are involved in pain modulation and processing, cognition, affective behavior, addiction and awareness [87]. A recent study described disturbances in white matter integrity in the insular cortex and in the parietal operculum [113]. It has to be noted however that not all studies found the same morphologic differences in the brains of MOH patients with migraine, including those comparing  scans before and after withdrawal [87, 114, 115].

Functional imaging has shown altered functional connectivity in pain processing areas, the mesocorticolimbic ‘reward’ system, the salience network, the fronto-parietal attention network, the default network and memory processing networks [87, 113, 115–118]. The mesocorticolimbic dopaminergic ‘reward’ system, characterized by structures such as the ventromedial prefrontal cortex (VMPFC), the nucleus accumbens and the substantia nigra/ventral tegmental area, seems to be affected in MOH, linking psychiatric characteristics such as dependence mechanisms and addictive components to the disorder [87, 117, 118].

It is noteworthy that, in MOH, changes of functional connectivity and structure may be reversible in some but not all regions and sometimes normalize after treatment [87]. By using [18F] fluorodeoxyglucose-Positron emission tomography (FDG-PET), it was detected how several pain processing regions in the brain were hypometabolic during medication overuse but recovered to normal metabolism after withdrawal. An exception to these findings was found in the OFC, a region linked with drug dependence and addiction. This region remained hypometabolic despite discontinuation of analgesics [119]. Other groups have confirmed changes in this region of interest. Reduction of gray matter volume in the OFC was correlated with headache days at follow-up, hereby exhibiting predictive capability in terms of poor response to treatment [112, 120]. Non-responders to withdrawal therapy seemed to have less gray matter in the OFC on their pre-detoxification scan and that there was a positive correlation of gray matter in the OFC with response to treatment [121]. Interestingly, in a longitudinal study, MOH patients with clinical improvement after withdrawal had a significant decrease of previously increased gray matter in the midbrain (PAG, nucleus cuneiformis), whereas patients without improvement did not [121]. Another group found that VMPFC dysfunction is reversible and might be attributable to headache, whereas dysfunction observed in the mid-brain dopaminergic areas (substantia nigra/ventral tegmental area) are probably long-lasting and related to drug overuse [116, 117]. In conclusion, the evidence provided in multiple studies shows that medication overuse causes changes to the CNS in people with an underlying susceptibility for progression. Changes in pain processing networks, dependence networks, sensitization and receptor density in the CNS presumably explain the clinical characteristics of the disorder.

## Treatment

### Education and prevention

MOH is often considered to be a preventable condition [122]. Instructing patients about the relationship between an excessive use of acute medications and headache progression is an important preventive measure. The results from multiple studies have shown that most MOH patients have little to no knowledge about excessive drug intake headache chronification. Many patients however received correct information, but often did not remember or had not fully understood the message [123–125]. As in other patients with chronic pain conditions, MOH patients seem mainly focused on the side effects related to the acute pain medications, including gastrointestinal bleeding, kidney damage, and liver impairment. They are often surprised when they learn that the excessive use of acute pain drugs might increase headache frequency, leading to MOH [126]. This is due to the fact that for many MOH patients the symptomatic medications are merely the drugs they need to relief their pain, and the only way that could bring relief to the impact on their lives [127].

According to this evidence, developing information campaigns and strategies to target patients at risk, preferably before MOH onset, represents an essential objective in headache medicine. In German headache centers, a brochure on medication overuse was effective in preventing the development of MOH in people with migraine and frequent medication use [128]. Primary care is the best setting for prevention and initial treatment of MOH, since most MOH patients consult their general practitioner (GP) for headache (80%) [129]. GPs can play a key role in providing patient education about medication use and modifiable risk factors, such as stress, daily smoking, physical inactivity, and obesity [82]. GPs are also capable of prescribing first-line headache prophylaxis in episodic patients when required.

MOH patients often bypass medical advice by using over-the-counter medication. A study recruited patients in pharmacies and found that only 14.5% were ever advised to limit intake frequency of acute headache treatments [130]. In a recent Swedish study investigating the knowledge of 326 pharmacists on headache treatment, only 8.6% demonstrated knowledge that overuse of all types of acute headache medications could lead to the development of MOH [131].

In 2016, the Danish national awareness campaign for MOH was conducted to reach the general public, GPs, and pharmacists. Online resources, print media, radio interviews, and a television broadcast were used to bring key messages such as overuse of pain medication can worsen headaches, pain medication should be used rationally, and medication overuse headache is treatable. The survey showed an increase in percentage of the public who knew about MOH [132].

### Withdrawal as the first phase of treatment

Despite the large controversies about whether medication overuse should be regarded as a cause or a consequence of headache chronification, to date, the worldwide consensus agrees that (ideally complete) withdrawal of acute painkilling drugs is the approach of choice for the acute management of MOH patients [133–135]. In a recent randomized controlled open-label trial, complete discontinuation  of acute medications came forward as the most effective detoxification program compared to restricted drug intake [136]. Drug discontinuation is advised in most headache treatment guidelines, including guidelines for primary care [137–139] . The crucial therapeutic aspect of withdrawal is that, on the one hand, it is an occasion for the physician to help the patient decrease or stop the use of acute medication, while potentially initiating a new preventive therapy. It is an opportunity for the patient to reconsider his or her headache history, to discover the link with medication overuse and to be guided by the physician in the process of withdrawal [140].

Drug discontinuation is performed variously in different headache clinics. In terms of timing, no studies have investigated the abrupt interruption versus the progressive cessation of the overused drugs, but it is widely agreed that for triptans, ergots, combination analgesics, simple analgesics, and NSAIDs the abrupt withdrawal is the treatment of choice, since these medications do not cause severe withdrawal symptoms [137]. On the opposite, a gradual drug reduction is the best option with barbiturates, benzodiazepines, and opioids [137]. Withdrawal symptoms (e.g. headache, nausea, vomiting, arterial hypotension, tachycardia, sleep disturbances, etc) generally last for 2–10 days. Seizures or hallucinations are rare, even in patients who are barbiturate abusers. The withdrawal phase is shorter in subjects who excessively use triptans [141].

Certain studies have demonstrated that simple information and advice may be enough to achieve headache improvement in many MOH patients [142, 143]. In the Brief Intervention for Medication-Overuse Headache (BIMOH) study, a sample of MOH patients received a brief intervention of education on medication overuse from their GPs. After 3 months, headache and medication days were reduced by 7.3 days/month, and chronic headache resolved in 50% of the cases [129]. The effectiveness of this brief intervention was confirmed at 6 month-follow up: headache and medication days were reduced by 5.9 days/month, and chronic headache resolved in 63% [144].

Deciding on the setting for withdrawal is a key point of MOH treatment. The choice between outpatient and inpatient withdrawal has to consider many factors, including the patient’s motivation, the duration of the overuse, the type of overused drugs, possible previous detoxification failures and comorbidities. An outpatient detoxification can be the preferred setting for highly motivated patients, with a short duration of overuse of simple analgesics, and whose everyday life make an inpatient withdrawal unsuitable [145]. Instead, inpatient withdrawal therapy is recommended for patients overusing more complex analgesics (such as opioids, tranquilizers, or barbiturates), long duration of overuse, previous failure to withdraw drugs as outpatients and in more complex clinical situations (e.g. psychiatric comorbidities) [137]. No standardized therapeutic protocol for medication withdrawal is accepted worldwide. Different strategies are employed in clinics such as intravenous hydration, rescue medications such as IV aspirin and IV dihydroergotamine, symptomatic drugs other than those overused, and drugs for withdrawal symptoms including antiemetics (e.g. metoclopramide), clonidine, benzodiazepines, and corticosteroids [36, 146–150]. Considering corticosteroids, there is low evidence for change in various headache outcome measures (i.e. use of rescue medication, days with severe or moderate headache, days without headache, headache days, and headache frequency) [151, 152]. Evidence in favor of inpatient withdrawal comes from an observational study showing statistically significant improvement of quality of life, depression and anxiety at 6-month follow-up [153]. Furthermore, it is recognized that a proper therapeutic approach to MOH requires a multistep and multidisciplinary program [154, 155]. The “COMOESTAS” consortium provided an expert consensus protocol in four centers from Europe and two centers in Latin American. The results show that after multiphasic and personalized treatment, two thirds of patients were no longer overusers and almost half reverted to an episodic headache syndrome over a six-month period [156].

### Prophylaxis

The initiation of preventative therapy is a fundamental therapeutic step to prevent episodic headache converting into a chronic condition. However, the question remains unresolved whether starting prophylactic treatment at the beginning of withdrawal or awaiting the effect of detoxification is the most effective approach. Certain authors recommend that in non-complicated MOH patients, the decision to start preventive treatment may be postponed for two to 3 months following withdrawal. On the contrary, patients who already have a high frequency of headaches before medication overuse and who have been previously treated with more than one preventive treatment, might need early prophylaxis [157]. Other clinicians believe that the detoxification can be effective without an immediate prophylaxis [126]. To date, as confirmed in a recent meta-analysis of randomized controlled trials on the effect of prophylactic therapies (i.e. valproate, nabilone, onabotulinumtoxinA, topiramate, amitriptyline), there is not a preventative drug that has demonstrated superiority to other therapies in a qualitative, appropriately designed study [152]. The results of randomized controlled trials with patients affected by chronic migraine and MOH suggest the use of onabotulinumtoxinA and topiramate without early discontinuation. However, the quality of the data is limited due to the fact that it is based on post hoc analysis [158]. A future role for monoclonal antibodies targeting the CGRP pathway is to be awaited [159]. Ultimately, the identification of proper prophylaxis should be driven by clinical history, comorbidity, contraindications and side effects of the possible drugs [126].

### Treatment of comorbidities

Comorbidities have important implications for the management of MOH in daily clinical practice. Co-existence of mood disorders may lead to poorer adherence of headache treatment, leading to unsuccessful headache treatment. Comorbid psychiatric disorders add to overall burden and reduced quality of life in headache patients and may lead to poorer outcomes after treatment. Therefore, screening patients for anxiety and depression, is important for clinical outcomes and for trials studying MOH. Lastly, attention for metabolic disturbances or unhealthy lifestyle behavioural aspects, such as obesity, smoking and inactivity, in daily practice is probably beneficial not only for general health but also for headache outcomes. As these are mostly modifiable factors, it is reasonable to discuss and treat these conditions accordingly.

## Prognosis

In general, overuse of acute treatment can lead to a poor prognosis of chronic headache and lower quality of life by itself [160]. The outcome for MOH patients withdrawing from their acute treatments has been reported in multiple studies. An accepted endpoint for good response to therapy is a ≥ 50% reduction from baseline headache frequency and/or headache index [161]. Successful withdrawal has been found in around 50–70% of MOH patients after 1 year [68, 162–170]. Retaining full withdrawal after 1 year was found to be a good predictor for long-term success [171, 172]. In studies with long-term evaluations up to 6 years, relapsing rates between 40 and 50% were found [163, 164, 173–177]. A successful withdrawal leads to a better response for prophylactic treatment, even in patients with little improvement in headache frequency [178]. Multiple predictors of relapse have been documented. Patients with tension-type headache have a higher relapse risk [162–164, 173, 179]. A longer duration of regular intake is a predictor for relapse [174, 180]. Patients who kept overusing medication in the long-term had a poor response to withdrawal therapy and had a higher frequency of chronic headache [171]. Risk factors for relapse in short-term (1 year) were: high number of acute treatments, smoking, alcohol consumption and return to overused drugs [181]. Patients withdrawn from triptans have a lower risk for relapse, while combined drug therapy had a higher relapse rate [163, 179, 182]. Codeine containing drugs, low self-reported sleep quality and high self-reported bodily pain are probable predictors for poor outcome after 1 yr [170].

## Debates in MOH

The idea of MOH is well-known and widespread in clinics worldwide. By using the operational criteria for MOH in the ICHD-3 classification, clinicians are able to diagnose MOH as early as first clinic visits in order to guide patients in cutting down the amount of frequently used analgesics.

The evidence in favor of the disorder MOH is substantial since global research has gradually improved our knowledge on the complexity of the disorder. Consistent observations from population-based longitudinal studies by headache experts in expertise centers worldwide, have established the entity of MOH in a considerable amount of headache patients. Research on the pathophysiological mechanisms is steadily unravelling the different processes involved with analgesic overuse in headache syndromes. Agreement in findings from imaging studies for entity-specific alterations in the brain have been published, although the amount of data is still limited and needs further research [86, 87]. Furthermore, the results from neuroimaging suggest that neuroplasticity exists and that specific imaging findings can be predictive for the outcome after withdrawal. Lastly, the field of genetics in MOH is in development, reaching out to a more personalized approach for MOH [159].

However, it is important to raise awareness on the current limitations to the state of literature on MOH. Questions need to be put forward on how to analyse the phenomenon of deteriorating headaches with the use of analgesics. Mostly, the disorder is seen in patients with pre-existing headache disorders and therefore analysing it as a complication to these conditions is reasonable. Given the potential for the onset of chronic headache after regular intake of analgesics for other medical conditions, MOH can be conceptualized as a secondary headache disorder. But reminding ourselves to patients who experience increasing headache severity and frequency without drug overuse, the overuse of analgesics can be seen as a mere epiphenomenon to the primary headache disorder, a cycling disorder with good and bad phases, for which treatment of the headache syndrome without detoxification is required [155, 183]. The scientific community has not yet come to the end of this discussion. The lack of robust evidence from high-quality, well-designed and large randomized controlled clinical trials on MOH is important in this analysis [184]. Withdrawal studies over the years have delivered evidence of moderate quality, mostly due to the lack of control groups, lack of randomization, difficulties in adequate blinding and often high dropout rates [158, 184]. Furthermore, evidence in favour of starting prophylactic treatment in MOH comes from post-hoc analysis without adequate power [134, 158]. One of the most critical aspects of MOH treatment concerns discontinuation of the symptomatic medication. This concept is installed by using observational data and specialist consensus, not on solid level of evidence from large and well powered randomized, blinded trials [137, 139]. Because of the huge burden of disease for patients, larger and high-quality interventional trials on the efficacy of treatments are needed [158, 184]. This is complicated by a significant limitation. For an individual patient, the existence of MOH can neither be proven nor invalidated due to the lack of pathognomonic clinical aspects or a clinical useful biomarker, and therefore studies will still rely on consensus criteria.

The diagnostic criteria for MOH in the international classification remain fuel for debate, even after three editions and multiple decades. The discussion whether MOH has a rightful place in the classification as a secondary headache disorder is interesting and relevant. The current ICHD-3 criteria do not denominate MOH to be a ‘transformed’ version of a primary headache disorder, but instead describe a concurrent medical problem occurring with an underlying headache disorder. The diagnosis is neither a definitive claim on the cause of a progressive headache disorder either. It has a more moderate approach to the occurrence of medication overuse than previous clinical criteria. Furthermore, the current classification uses clinical features that do not touch upon on the underlying neurobiological processes and has rigid elements such as the cut-off of 15 days per month. These elements may need to be revisited when new evidence becomes available in the future [23].

Finally, different authors discussed the previous, current and possible future terminology used in the field of MOH. In terms of semantics, the term “medication-overuse headache” was challenged by Solomon et al*.* in 2011 [185]. MOH has a potential stigmatizing and (self-) blaming message to patients that can put pressure on a good patient-physician relationship. Appellations as “iatrogenic headache” and “medication-overtreatment headache” have a potential to lay blame on healthcare providers [184]. On the other hand, terminology such as “medication misuse headache”, “medication abuse headache”, “drug abuse headache” also carry a clue of leaving patients responsible for the development of the situation. Hence Solomon et al analyzed possible mechanism-based definitions, for instance “medication-induced headache”, “feed-forward headache”, “drug-transformed (or amplified) headache” and suggested implicating the term “medication-adaptation headache” as the most appropriate [185].

To summarize, after analyzing the literature on MOH, it is our understanding that for clinicians in daily practice, the evaluation of the frequency and quantity of use of analgesics in patients with headache syndromes is a key component of medical assessment in headache disorders. Side effects of analgesic overuse must be actively evaluated and treated accordingly. However, MOH is not to be diagnosed rapidly without further intellectual perseverance, since a false-positive diagnosis of MOH can lead to misdiagnosis. Other secondary causes of headache may lead to headache progression in conjunction with medication overuse. We therefore state that a critical appraisal of the entity of MOH is required in every individual patient. A thorough clinical approach with exact history taking to detect temporal relationships, and clinical examination focused on neurological deficits, remain the foremost necessary assets for clinicians in the absence of accurate technical tools.

## Conclusion

Research in MOH is moving forward and is discovering the mechanisms underlying headache progression and medication overuse. Whether MOH is a definitive distinct entity, a complication in the pathophysiology of primary headache disorders or an epiphenomenon in the natural course of headache disorders is still up for debate. Since methodology is improving and world-wide collaborative efforts are being established, it is clear that high-quality research will help us to resolve multiple questions stated above. Ultimately, by making scientific progress, we are hopeful that new evidence will help clinicians to make the right choices for patients suffering highly disabling headaches and comorbidities.

## Abbreviations

5HT-2A: Serotonin 2A
BIMOH: Brief Intervention for Medication-Overuse Headache
CGRP: Calcitonin-gene related peptide
CM: Chronic migraine
CSD: Cortical spreading depression
CTTH: Chronic tension-type headache
EM: Episodic migraine
FDG-PET: [18F] fluorodeoxyglucose-Positron emission tomography
GBD: Global Burden of Disease
GP: General practitioner
HADS: Hospital Anxiety and Depression Scale
ICHD: International Classification of Headache Disorders
ICHD-2: Second Edition of the International Classification of Headache Disorders
ICHD-3: Third Edition of the International Classification of Headache Disorders
MOH: Medication-overuse headache
NSAID: Non-steroidal anti-inflammatory drug
OCD: Obsessive-compulsive disorder
OFC: Orbitofrontal cortex
OR: Odds ratio
PAG: Periaqueductal gray
RR: Relative risk
SAMOHA: Sodium Valproate in Medication Overuse Headache Treatment
VMPFC: Ventromedial prefrontal cortex
